# Blocking NHE1 stimulates glioma tumor immunity by restoring OXPHOS function of myeloid cells

**DOI:** 10.7150/thno.50150

**Published:** 2021-01-01

**Authors:** Md Nabiul Hasan, Lanxin Luo, Dawei Ding, Shanshan Song, Mohammad Iqbal H. Bhuiyan, Ruijia Liu, Lesley M. Foley, Xiudong Guan, Gary Kohanbash, T. Kevin Hitchens, Maria G. Castro, Zhongling Zhang, Dandan Sun

**Affiliations:** 1 Department of Neurology and Pittsburgh Institute for Neurodegenerative Disease, University of Pittsburgh, Pittsburgh, PA 15213.; 2 Animal Imaging Center, University of Pittsburgh, Pittsburgh, Pennsylvania, 15213.; 3 Department of Neurological Surgery, University of Pittsburgh, Pittsburgh, PA 15213.; 4 Department of Neurobiology, University of Pittsburgh, Pittsburgh, Pennsylvania, 15213.; 5 Department of Neurosurgery, University of Michigan Medical School, Ann Arbor, MI, USA.; 6 Department of Neurology, First Affiliated Hospital, Harbin Medical University, Harbin, Heilongjiang, China.; 7 Veterans Affairs Pittsburgh Health Care System, Geriatric Research, Educational and Clinical Center, Pittsburgh, Pennsylvania.

**Keywords:** anti-PD-1 therapy, glycolysis, immunosuppression, temozolomide, tumor-associated microglia/macrophage

## Abstract

**Background:** Immunosuppressive tumor microenvironment (TME) in glioblastoma (GBM) is one of the contributing factors for failed immunotherapies. Therefore, there is an urgent need to better understand TME and to identify novel modulators of TME for more effective GBM therapies. We hypothesized that H^+^ extrusion protein Na/H exchanger 1 (NHE1) plays a role in dysregulation of glucose metabolism and immunosuppression of GBM. We investigated the efficacy of blockade of NHE1 activity in combination with temozolomide (TMZ) therapy in increasing anti-tumor immunity.

**Methods:** Mouse syngeneic intracranial glioma model was used to test four treatment regimens: DMSO (Vehicle-control), TMZ, NHE1 specific inhibitor HOE642, or TMZ+HOE642 (T+H) combination. *Ex vivo* ^1^H/^19^Fluorine magnetic resonance imaging (MRI) with cell tracking agent Vsense was performed to monitor the infiltration of glioma-associated microglia/myeloid cells (GAMs). Glucose metabolism and transcriptome profiles were analyzed by Seahorse analyzer and bulk RNA-sequencing. The impact of selective *Nhe1* deletion in GAMs on sensitivity to anti-PD-1 therapy was evaluated in transgenic *NHE1* knockout (*KO*) mice.

**Results:** Among the tested treatment regimens, the T+H combination therapy significantly stimulated the infiltration of GAMs and T-cells; up-regulated Th1 activation, and mitochondrial oxidative phosphorylation (OXPHOS) pathway genes, increased glucose uptake and mitochondrial mass, and decreased aerobic glycolysis in GAMs. Selective deletion of *Nhe1* in Cx3cr1^+^ *Nhe1 KO* mice increased anti-tumor immunity and sensitivity to TMZ plus anti-PD-1 combinatorial therapy.

**Conclusions:** NHE1 plays a role in developing glioma immunosuppressive TME in part by dysregulating glucose metabolism of GAMs and emerges as a therapeutic target for improving glioma immunity.

## Introduction

The highly malignant brain tumor, glioblastoma (GBM) has a dismal median patient survival of ~15 months, despite the current aggressive standard therapy of surgical resection followed by radiotherapy and temozolomide (TMZ) chemotherapy 1. Recent advances in immunotherapy for curing other cancers show no superior effects in increasing progression-free survival in glioma patients 2. Immunosuppressive tumor microenvironment (TME) in GBM is one of the contributing factors for failed immunotherapies 3. Therefore, there is an urgent need to better understand TME and to identify novel modulators of TME as new therapeutic targets for GBM.

Glioma-associated microglia and myeloid cells (GAMs) constitute about one-third of GBM tumor mass and promote tumor growth through bidirectional interactions with glioma cells 4-6. The TME features low glucose, oxygen, and high acidity 7, resulting from aerobic glycolysis (Warburg effect) of highly proliferative cancer cells to meet their high nutrient demand, despite the supply of oxygen and fully functioning mitochondria 8. Likewise, GAMs change their cellular metabolism that shapes their functional phenotypes. GAMs employ aerobic glycolysis for proliferation and survival 9 and polarize towards immunosuppressive phenotypes by increased production of IL-10, arginase-1, defective antigen presentation, and increased phagocytosis 10, 11. Additionally, increased glucose uptake of tumor cells makes tumor-infiltrating T-cells less capable of sufficient glucose uptake for its anti-tumor effector and memory function 7. These changes collectively contribute to an immunosuppressive TME and immunotherapy resistance in GBM 12. However, how to rewire glucose metabolism of GAMs and stimulate an immunogenic TME in GBM remains unknown and is a new frontier in research for improving immune checkpoint blockade therapy for GBM.

Na/H exchanger 1 (NHE1) protein is the main H^+^ efflux mechanism in maintaining alkaline pH_i_ in GBM, which is the driving force for glycolytic metabolism 13. Our recent studies indicate that NHE1 protein is involved in regulating GBM immunosuppressive TME because combining anti-PD-1 immune checkpoint blockade with NHE1 inhibitor HOE642 with or without TMZ therapy increased immunity in an immunogenic mouse syngeneic intracranial GBM model 14, 15. Bioinformatic analysis of the Chinese Genome Glioma Atlas (CGGA) cohort showed a positive correlation of *SLC9A1* (alias for NHE1) gene with the metagene score of monocyte and macrophages 15. However, the underlying molecular and cellular mechanisms of the role of NHE1 in developing immunosuppressive TME remain unknown.

New research emerges that dysregulation of metabolism and anti-tumor functions of macrophages and T-cells are intertwined 16, 17. Therefore, metabolic reprograming of macrophages and T-cells is new therapeutic strategies against GBM. In this study, we directly investigated the role of blocking NHE1 function in tumor-infiltrating immune cells and the impact on their glucose metabolism and immunosuppression. Using high-resolution proton and fluorine (^1^H/^19^F) magnetic resonance imaging (MRI), we observed that pharmacological inhibition of NHE1 protein activity combined with TMZ therapy increased proinflammatory myeloid cells and T-cell infiltration into nonimmunogenic GBM tumors. This in part resulted from reduced aerobic glycolysis and increased mitochondrial oxidative phosphorylation metabolism (OXPHOS), and anti-tumor polarization of GAMs. Importantly, using genetically engineered mice, selective deletion of *Nhe1* from Cx3cr1^+^ microglia/myeloid cells elevated CD4^+^PD-1^+^ and CD8^+^PD-1^+^ tumor infiltration in response to TMZ monotherapy. This immunogenic TME sensitized tumors to TMZ plus anti-PD-1 combination therapy. Our new findings demonstrate that NHE1 protein promotes immunosuppressive glioma TME in part via stimulating glucose metabolism and emerges as a novel target for improving immunotherapy.

## Methods

Reagents and chemicals are described in the supplementary information.

### Animals

All animal experiments were approved by the University of Pittsburgh Institutional Animal Care and Use Committee (IACUC) and performed in accordance with the National Institutes of Health Guide for the Care and Use of Laboratory Animals.

### Cell cultures

Immunogenic mouse GBM GL26 cells were obtained from Prof. Vadlamudi of UT Health, San Antonio and were cultured in DMEM/HEPES medium supplemented with 10% heat-inactivated FBS, 2 mM L-glutamate, 1% sodium pyruvate, 1% penicillin/streptomycin as adherent culture at a density of 5 × 10^5^ cells/75 cm^2^. Non-immunogenic mouse SB28-GFP glioma cells (5 × 10^4^ cells/75 cm^2^) were derived and cultured as described previously 14. Cultures were passaged twice in a week and passages of 8-25 were used. Both cell lines were authenticated by single tandem repeat profiling (IDEXX BioResearch, Columbia, MO). PCR analysis was performed to confirm the absence of mycoplasma infection in all cell cultures.

### Orthotopic glioma model and drug treatment

With stereotactic tumor implantation as described previously 14, 0.5 × 10^5^ SB28-GFP cells or 1 × 10^5^ GL-26 cells (in 2 μL of serum-free DMEM) were injected in the right striatum of C57BL6/J wild type mice (Jackson Laboratories, female, 6-10 weeks old) with coordinates from bregma (+0.5 mm AP, +2.1 mm ML, and -3.2 mm DV) in 4 min at a rate of 0.5 μL/min, using a micro-pump injector and a 5-μl Hamilton syringe equipped with a 33-gauge needle. Cells were allowed to settle for 5 min and the needle was withdrawn slowly. The hole was sealed with sterile bone wax and the incision was closed with sterile tissue adhesive. On the 5^th^ day of post- implantation (d.p.i.), mice randomly received either vehicle (Veh) control (25% dimethyl sulfoxide, DMSO, 1.25 ml/kg/day, i.p), TMZ (50 mg/kg/day in DMSO, i.p.), HOE-642 (0.3 mg/kg/day in DMSO, i.p) or T+H combination for five consecutive days.

### ^1^H and ^19^F MRI

At 13 d.p.i, C57BL6/J glioma bearing mice received an intravenous injection of V-Sense (Celsense, Pittsburgh, PA), a perfluorocarbon (PFC)-based nanoemulsion for ^19^F MRI cell tracking of *in-vivo* labeled immune cells. VS-1000H DM-red is a dual-mode reagent and has a covalently linked dye for optical immunofluorescence co-registration. Mice were given a dose of 5 mL/kg body weight via tail vein. At 48 h post-injection, mice were transcardially perfused and fixed with 4% paraformaldehyde. *Ex vivo* MRI was performed using a 7T/30-cm AVIII spectrometer (Bruker Biospin, Billerica, MA) equipped with a 12 cm gradient set and using a 35 mm ^1^H/^19^F dual-resonance birdcage RF coil and Paravision 6.0.1. A 5% solution of V-Sense was placed below the mouse that was used for ^19^F RF pulse calibration and concentration reference standard. Axial ^1^H anatomical images of the head were collected using a T_2_-weighted RARE sequence with the following parameters: repetition time (TR)/echo time (TE) - 4000/80 ms, 30 × 30 mm field of view, 192 × 192 acquisition matrix, 13 slices with a slice thickness of 1 mm, 12 averages, and a RARE factor of 8. Following the anatomical scans, ^19^F images were acquired using the same geometry for image co-registration except at lower resolution with higher signal averaging to increase sensitivity to the dilute PFC label (TR/TE = 2000/30, 96 × 96 matrix and 256 averages) 18. ^19^F images were rendered in “hot iron” pseudo-color and overlaid on the corresponding ^1^H images to place PFC-labeled immune cells in the anatomical context. Mouse tails were screened for ^19^F signal to determine the efficacy of the injections. One mouse was excluded from analysis due to a failed tail vein injection, evidenced by an excessive ^19^F signal in the tail. Quantification of ^19^F-signals was performed using Voxel Tracker (Celsense). ROIs were manually drawn around fluorine signals in the brain. The total fluorine signal for each ROI was summed and quantified using signal from a concentration reference standard in the imaging field of view. For individual mice, the total ^19^F signal was divided by tumor volumes, measured from the T_2_-weighted anatomical images.

### Immunofluorescence staining

Immunostaining of post-^1^H/^19^F MRI brain sections has been described in the supplementary methods.

### Flow cytometry

Flow cytometric profiling has been described in the supplementary methods. Briefly, GAMs were stained with anti-mouse APC-CD11b, BV510-CD45, PE-Ym1, eFluor 450-CD16/32, and T-cells were stained with Percp/Cy5.5-CD8a, Apc/Cy7-CD4, APC-CD25, PE-FoxP3, APC-IFNγ, and PE-GZMb.

### Extracellular flux analysis of GAMs

Seahorse glycolysis and mitochondrial stress tests were performed as per the Manufacturer's Instruction (Agilent Technologies). CD11b^+^ GAMs were isolated from GBM tumor tissues using magnetic-activated cell sorting (MACS). The single-cell suspension was resuspended in PBS containing 0.5% FCS and incubated with CD11b microbeads. The MACS separation was carried out according to the manufacturer's instruction. Cells were seeded in XF96 well plate at 1 × 10^5^ cells/well in DMEM base media. The extracellular acidification rate (ECAR) and oxygen consumption rate (OCR) were assessed with the addition of the following compounds, ECAR measurement: (i) 10 mM Glucose, (ii) 2 µM Oligomycin, (iii) 100 mM 2-deoxyglucose (2-DG). OCR measurement: (i) 2 µM Oligomycin, (ii) 1 µM Carbonyl cyanide-4-(trifluoromethoxy) phenylhydrazone, (iii) 1 µM Antimycin A + Rotenone. Measurements were done using an XF96 extracellular flux analyzer and results were analyzed using Wave v.2.2.0 software.

### RNA-seq of GAMs and bioinformatics analysis

CD11b^+^ GAMs (1 × 10^6^ Cells/sample) were isolated from glioma tumors by MACS and verified by flow cytometry. Total RNA was extracted using the RNeasy Mini Kit (Qiagen), and the library was prepared using the Illumina TruSeq RNA Library Prep Kit v2 (Illumina, USA), as instructed by the manufacturers. The libraries were quantified and subjected to sequencing on an Illumina Novaseq 6000 platform with 150 bp paired-end chemistry. Reads were mapped to a mouse genome (mm10) using the STAR aligner with default setting and differential gene analysis was conducted using DESeq2 in Partek Flow 8.0 software (Partek, USA). Gene expression levels were obtained through the count of total exon reads for statistical analysis 19. Enrichment analysis of DEGs was performed using Ingenuity Pathway Analysis (IPA®, QIAGEN Redwood City, www.qiagen.com/ingenuity) as described previously 20. IPA software compares the total number of occurrences of the genes in the provided dataset with their database by using computational algorithms and employs Fisher's exact test to detect significant enrichment.

### qRT-PCR of tumor isolated CD11b^+^ GAMs

RNA isolation from CD11b^+^ GAMs and quantitative RT-PCR has been described in the supplementary methods.

### Selective Nhe1 deletion in Cx3Cr1-Cre^ERT2+/-^; Nhe1^f/f^ mice

Heterozygous *Nhe1^f/f^* mice with one LoxP site inserted in the intron upstream of exon 5 and the other LoxP site in the intron downstream of exon 5 were developed as described recently 21. *Cx3cr1-Cre^ERT2^*transgenic mouse line (C57BL/6J; 129sv background) was crossbred with *Nhe1^f/f^* mice to generate *Cx3Cr1-Cre^ERT2+/-^*; *Nhe1^f/f^* mice and genotypes were determined by PCR analysis of DNA as described recently 22. At postnatal day 40-60 (P40-60), implantation of SB28 tumor was performed and tamoxifen (75 mg/kg/day, ip.) was administered for five consecutive days in* Cx3Cr1^CreET2r-/-^; Nhe1^f/f^* mice (*Con***)*** or Cx3Cr1-Cre^ERT2+/-^*; *Nhe1^f/f^* mice (*Nhe1 KO*) at 2-5 days post glioma injection (d.p.i.). For immune checkpoint blockade therapy, *Con* or *Nhe1 KO* mice were treated with IgG2a or anti-PD-1 (10 mg/kg/day, i.p.) at 10, 12, and 14 d.p.i. Mice were treated with TMZ (50 mg/kg/day in DMSO, i.p.) starting 5 d.p.i. for five consecutive days. Animal survival test was conducted based on a 20% reduction of body weight as the humane endpoint 14.

### Statistical analysis

All statistical analyses were performed using Graphpad Prism 7.0 software. Data were represented as mean ± standard error. Statistical significance was determined by analysis of variance followed by Bonferroni's multiple comparisons. Mouse median survival was evaluated using Kaplan-Meier analysis and significance was calculated by the log-rank test. Values of *p* < 0.05 were considered statistically significant. The number of animals used is discussed in each figure.

## Results

### Pharmacological blockade of NHE1 in combination with TMZ therapy increases infiltration of immune cells

**Figure 1:**
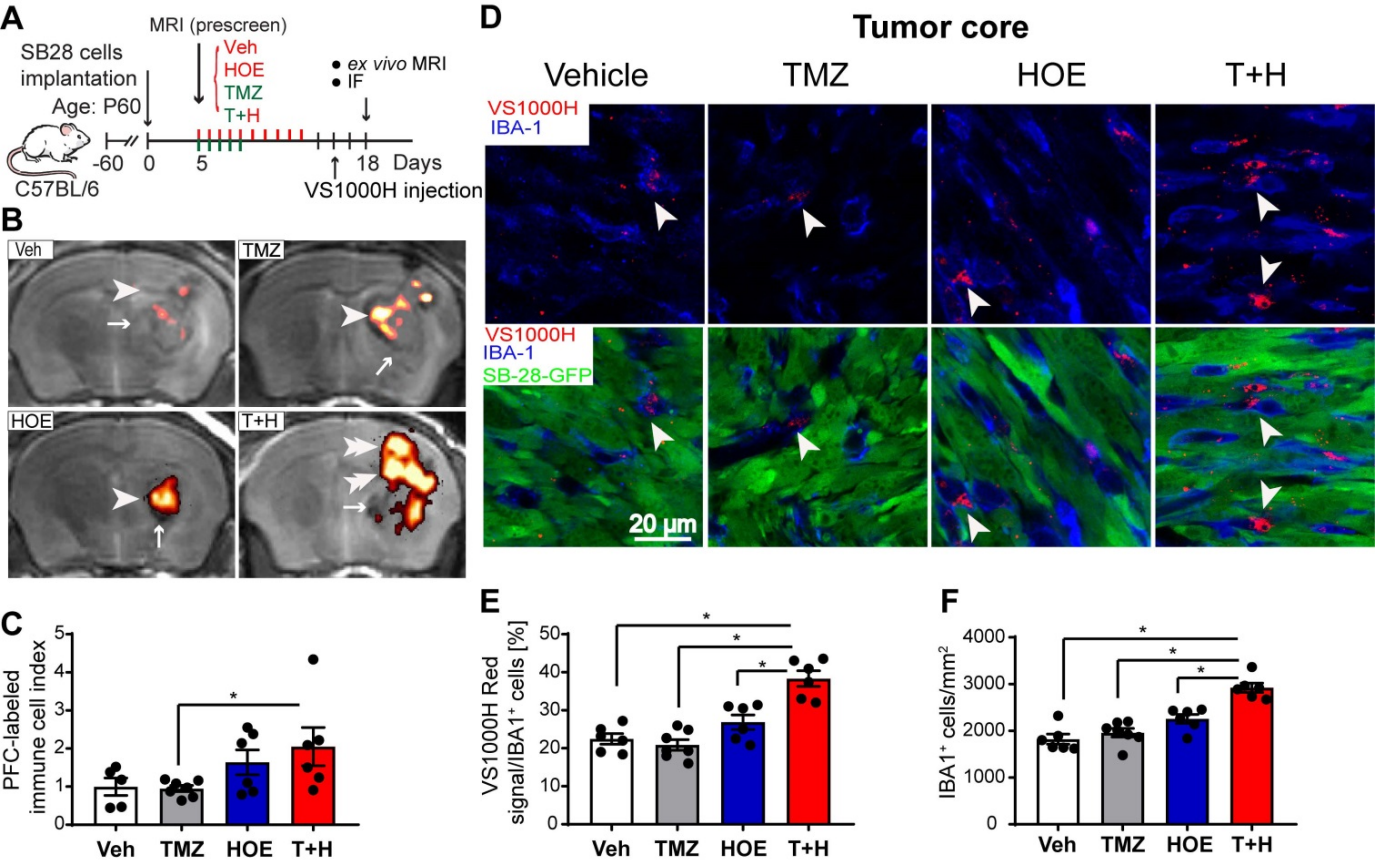
** Pharmacological inhibition of NHE1 in combination with TMZ increase immune cell infiltration determined by *ex vivo*^1^H/^19^F MRI and immunostaining.** (**A**) Experimental protocol showing that SB28-GFP cell implanted mice at 5 d.p.i. received either vehicle (PBS-DMSO), HOE642 (H), TMZ (T), or T+H combination treatments. VS1000H DM red emulsion was injected via tail vein on 16 d.p.i. and at 18 d.p.i mice were sacrificed to perform *ex vivo* MRI. (**B-C**) Representative images of ^1^H/^19^F MRI tracking for GAMs. ^19^F MRI (pseudocolor, arrowheads) over greyscale anatomical image (arrow). Immune cell infiltration index was computed as ^19^F signal divided by tumor volume and data were normalized to the control. (**D**) Representative confocal images of perfluorocarbon (VS1000H, Texas Red, arrowheads) loaded GAMs and IBA1 staining of brain sections collected post-MRI from the same cohort of mice used in panel (A-C). (**E**) Summary data of IBA-1 cells loaded with VS1000H dye. (F) Summary data of IBA-1^+^ cells infiltrated in the tumor core. Data are mean ± SEM, n = 5-7 mice/group. *p < 0.05 vs indicated.

The PFC contrast agent is non-toxic and is readily taken up by immune cells as foreign particles, the excess is eliminated via hepatic and renal mechanisms 18. An advantage of using PFC nanoemulsions for MRI cell tracking is that organic fluorine is not normally found in the body and ^19^F MRI can unambiguously detect PFC-labeled cells in the absence of a background signal. Labeled cells can then be placed into an anatomical context with typical ^1^H MRI (**Fig. 1A-B**). Thus, the PFC ^19^F MRI signal directly reflects the density of the PFC-labeled infiltrated immune cells (**Fig. 1C**). As shown in **Supplementary Fig. 1**, TMZ monotherapy decreased ^19^F signal intensity by ~30%, and HOE642 monotherapy or T+H combination therapy increased ^19^F signal by ~47% and ~60%, respectively. However, these changes did not reach statistical significance. Therefore, to study the association of immune cell infiltration with tumor size, an infiltration index of immune cells was computed as ^19^F signal/tumor volume normalized to the mean of the vehicle-control (**Fig. 1C**). Similar indices were detected in the Vehicle treated- and the monotherapy-treated tumors (with NHE1 inhibitor HOE642 or TMZ). However, the T+H combination therapy exhibited a ~2.5-fold increase in the immune cell infiltration index (**Fig. 1C**). To determine the localization of Vsense dye in myeloid cells, we analyzed the presence of the VS1000H DM (Texas red dye) in IBA-1^+^ myeloid cells. As shown in **Fig. 1D,** the VS1000H DM (Texas red) signals were detected in the IBA-1^+^ cells (**arrowhead**). Consistent with the ^1^H/^19^F MRI images, TMZ or HOE642 monotherapy did not change the number of IBA-1^+^/PFC-Texas Red labeled cells (either in the tumor core or tumor border). But, a ~2-fold increase in IBA-1^+^/VS1000H DM-red labeled cells were found in the T+H treated tumor core (**arrowhead, Fig. 1D-E,** *p* < 0.05). In addition, the T+H treatment significantly increased overall IBA-1^+^ cell numbers (**Fig. 1F**, *p* < 0.05). Similar findings were observed in the tumor border areas, with a ~1.5-fold increase in the IBA-1^+^/VS1000H DM-red labeled cells in the T+H group (**arrowhead, Fig. S2A-B**, *p* < 0.05). This change was accompanied with increased tumor-infiltrated CD8^+^ T-cells and their cell volume (**arrowhead, Fig. S3A-D**, *p* < 0.05). The larger size of CD8^+^ T-cells is a feature associated with proliferative and activated T-cells 23. Collectively, these findings suggest that the T+H combination therapy stimulates tumor innate immunity. This is consistent with our recent report that the T+H combination therapy reduced the infiltration of CD11b^+^/Gr1^+^ MDSC into SB28 glioma tumors 14.

### Pharmacological blockade of NHE1 protein in combination with TMZ therapy increases GAMs glucose uptake and mitochondrial OXPHOS

**Figure 2:**
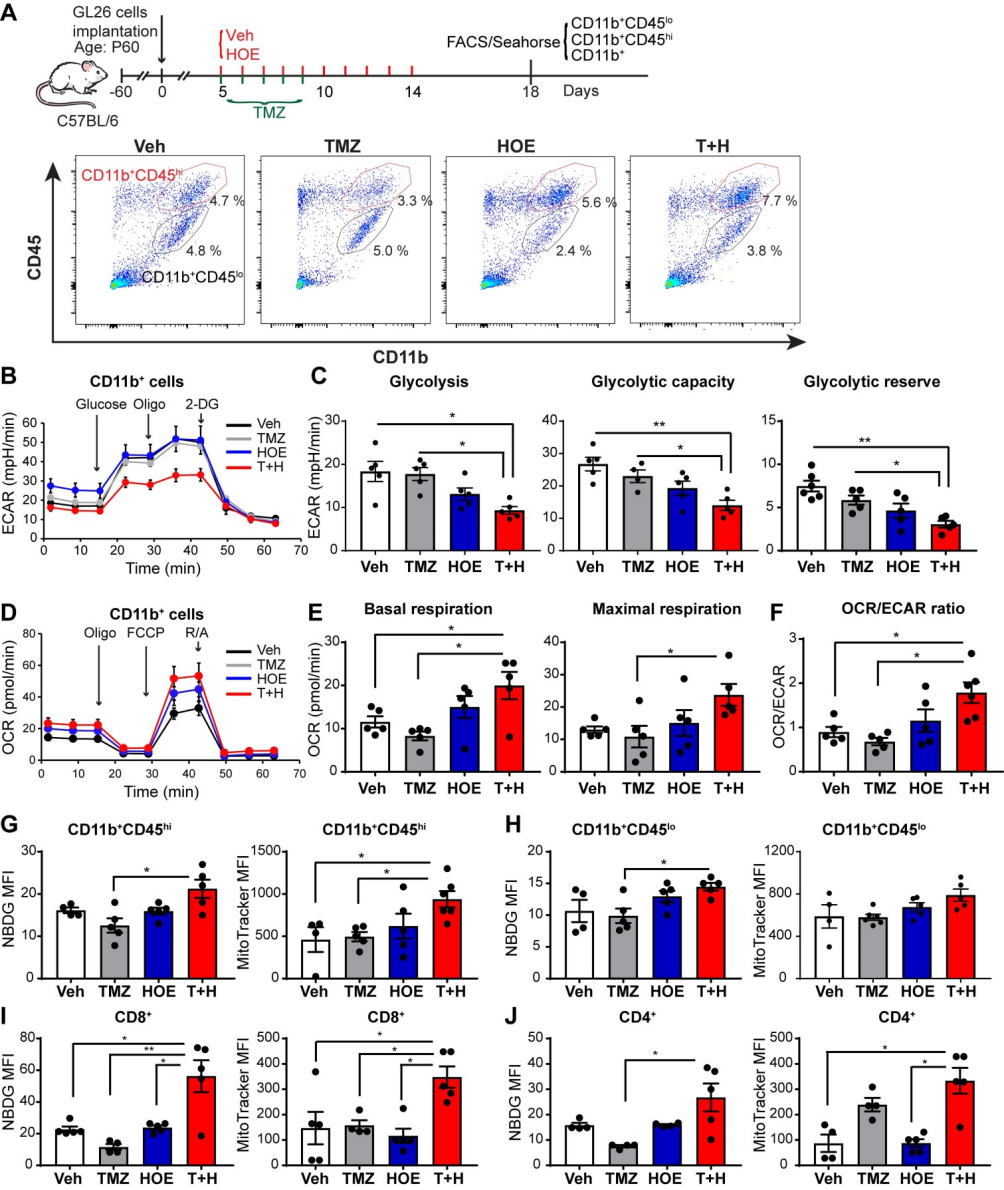
** Pharmacological inhibition of NHE1 in combination with TMZ increase glucose uptake and mitochondrial respiratory function in GAMs.** (**A**) Experimental protocol showing that GL26 cell implanted mice at 5 d.p.i. received either vehicle (PBS-DMSO), HOE642 (H), TMZ (T), or T+H combination treatments. Flow cytometric analysis or Seahorse XFe96 analysis was subsequently performed. (**B-F**) Extracellular acidification rate (ECAR) and oxygen consumption rate (OCR) were measured in tumor-infiltrated CD11b^+^ cells. (**G-H**) 2-NBDG uptake and mitochondrial Mitotracker staining of tumor infiltrated microglia (CD11b^+^/CD45^low-medium^) and myeloid (CD11b^+^/CD45^high^) cells. (**I-J**) 2-NBDG uptake and Mitotracker staining of tumor-infiltrated CD8^+^ and CD4^+^ T-cells. Data are mean ± SEM. n = 4-5 mice/group. *p < 0.05, **p < 0.01 vs indicated.

We speculated that the pharmacological blockade of NHE1 protein changed the immunosuppressive functions of GAMs via altering their glucose metabolism. First, we assessed changes of glycolysis and mitochondrial OXPHOS of GAMs from GL26 tumor following four regimen treatments, by measuring extracellular acidification rate (ECAR) and oxygen consumption rate (OCR) of CD11b^+^ cells in the seahorse extracellular flux assay (**Fig. 2A**). Monotherapy with NHE1 inhibitor HOE642 or TMZ did not change ECAR nor OCR of GAMs, compared to the Veh-control treatment. Interestingly, GAMs from the T+H regimen showed a 2-fold reduction of ECAR (**Fig. 2B-C,** *p* < 0.05) and ~2-fold increase of OCR, with a significantly increased ratio of OCR/ECAR (**Fig. 2D-F,** *p* < 0.05). These data indicate that GAMs from the T+H regimen show increased OXPHOS and reduced glycolysis and are more dependent on mitochondrial respiration.

To further investigate mitochondrial function, we measured glucose uptake and mitochondrial glucose metabolism of both CD11b^+^/CD45^low-med^ and CD11b^+^CD45^hi^ myeloid cell population (**Fig. 2A**). The efficacy of separating these two populations has been previously validated by probing for microglial specific marker P2RY12 expression in the CD11b^+^/CD45^low-med^ population 14. As shown in **Fig. 2G-H,** the pharmacological blockade of NHE1 protein with HOE642 did not change glucose uptake (determined by 2-NBDG) or mitochondrial mass (mitotraker fluorescent intensity) in either CD11b^+^CD45^hi^ myeloid cell or CD11b^+^CD45^low-med^ microglia populations. However, the T+H combination therapy significantly stimulated glucose uptake by ~1.5-fold in both populations (**Fig. 2G-H,** *p* < 0.05). Compared to the Veh- or TMZ-therapy, the T+H combination therapy increased the mitochondrial mass in the CD11b^+^CD45^hi^ myeloid cells by ~ 2-fold (**Fig. 2G**, *p* < 0.05). Taken together, these data further suggest that the T+H regimen rewires glucose metabolism of GAMs by increasing glucose uptake, mitochondrial mass, mitochondrial OXPHOS, and concurrently reduced glycolysis. Similar findings on elevated glucose uptake and mitochondrial mass were detected in the tumor-infiltrating CD8^+^ or CD4^+^ T-cells with the T+H combination therapy (**Fig 2I-J,** *p* < 0.05). These data further support our hypothesis.

**Figure 3:**
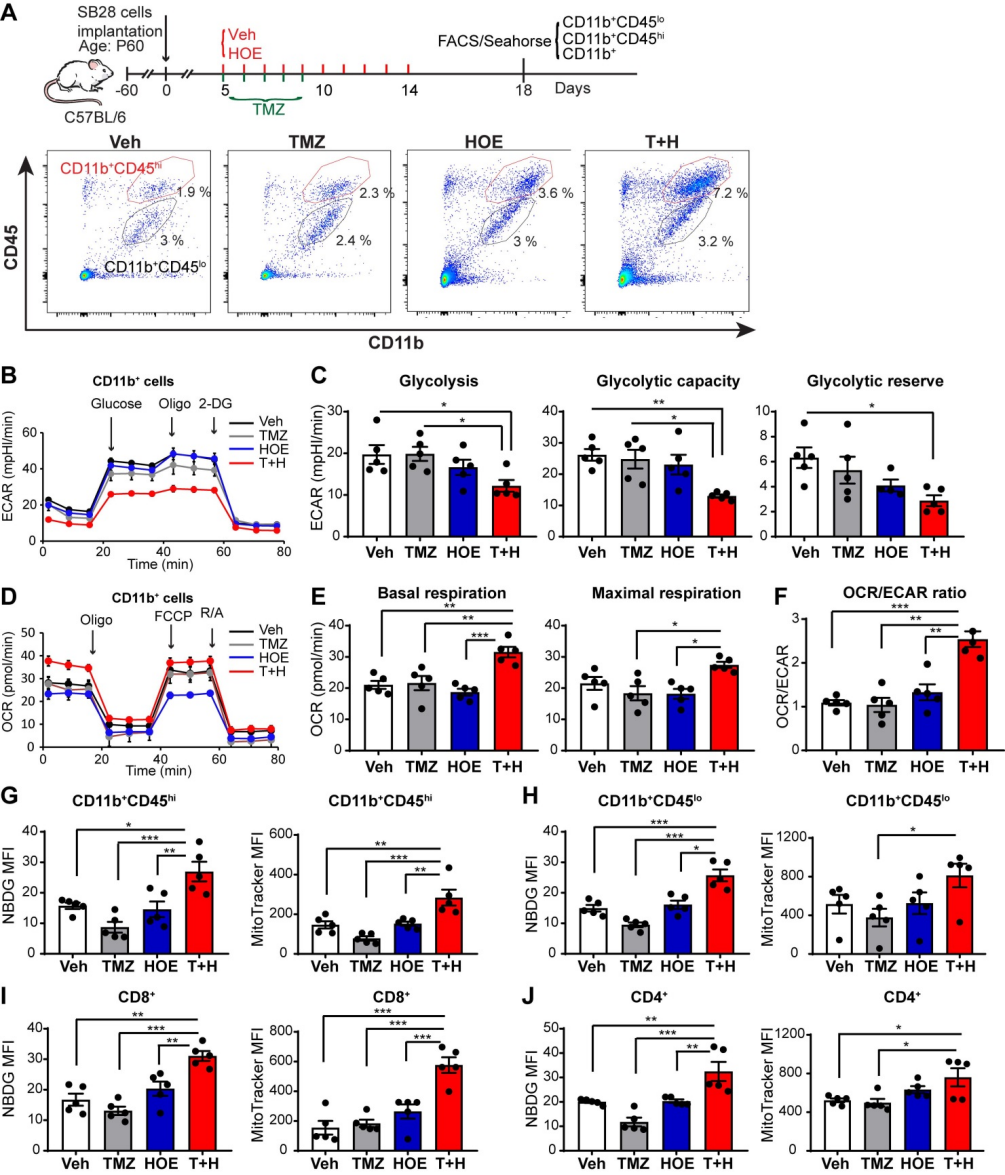
** Pharmacological inhibition of NHE1 in combination with TMZ increase glucose uptake and mitochondrial respiratory function in SB28-GFP tumor-infiltrating GAMs.** (**A**) Experimental protocol showing that SB28-GFP cell implanted mice at 5 d.p.i. received either vehicle (PBS-DMSO), HOE642 (H), TMZ (T), or T+H combination treatments. Flow cytometric analysis or Seahorse XFe96 analysis was subsequently performed. (**B-F**) Extracellular acidification rate (ECAR) and oxygen consumption rate (OCR) were measured in tumor-infiltrated CD11b^+^ cells. (**G-H**) 2-NBDG uptake and mitochondrial Mitotracker staining of tumor infiltrated microglia (CD11b^+^/CD45^low-medium^) and myeloid (CD11b^+^/CD45^high^) cells. (**I-J**) 2-NBDG uptake and Mitotracker staining of tumor-infiltrated CD8^+^ and CD4^+^ T-cells. Data are mean ± SEM. n = 5 mice/group. *p < 0.05, **p < 0.01, ***p < 0.001 vs indicated.

We also assessed changes of glycolysis, mitochondrial OXPHOS, and mitochondrial function of GAMs from poorly immunogenic SB28-GFP tumors following four regimen treatments (**Fig. 3A**). Similar to GL26 glioma, monotherapy with NHE1 inhibitor HOE642 or TMZ did not change ECAR nor OCR of GAMs, compared to the Veh-control treatment. GAMs from the T+H regimen showed ~1.6-fold reduction of ECAR (**Fig. 3B-C,** *p* < 0.05) and ~1.5-fold increase of OCR, with a significantly increased ratio of OCR/ECAR (**Fig. 3D-F,** *p* < 0.05). These data strongly support our conclusion that GAMs from the T+H regimen show increased OXPHOS and reduced glycolysis and are more dependent on mitochondrial respiration. Moreover, the pharmacological blockade of NHE1 protein with HOE642 did not change glucose uptake or mitochondrial mass in either CD11b^+^CD45^hi^ myeloid cell or CD11b^+^CD45^low-med^ microglia populations (**Fig. 3G-H**). However, the T+H combination therapy significantly stimulated glucose uptake by ~ 2.0-fold in both populations (**Fig. 3G-H**; *p* < 0.01). Compared to the Veh- or TMZ-therapy, the T+H combination therapy increased the mitochondrial mass in the CD11b^+^CD45^hi^ myeloid cells and CD11b^+^CD45^low-medium^ cells by ~1.9-fold and ~1.5-fold respectively (**Fig. 3G-H**; *p* < 0.01). Taken together, these data further suggest that the T+H regimen rewires glucose metabolism of GAMs by increasing glucose uptake, mitochondrial mass, mitochondrial OXPHOS, and concurrently reduced glycolysis. Similar to GL26 glioma, elevated glucose uptake and mitochondrial mass were detected in the tumor-infiltrating CD8^+^ or CD4^+^ T-cells with the T+H combination therapy (**Fig 3I-J,** *p* < 0.05). These data demonstrate that NHE1 is involved in maintaining the immunosuppressive function of GAMs via altered glucose metabolism.

### Pharmacological blockade of NHE1 protein in combination with TMZ therapy stimulates OXPHOS signaling genes in the GAMs

**Figure 4:**
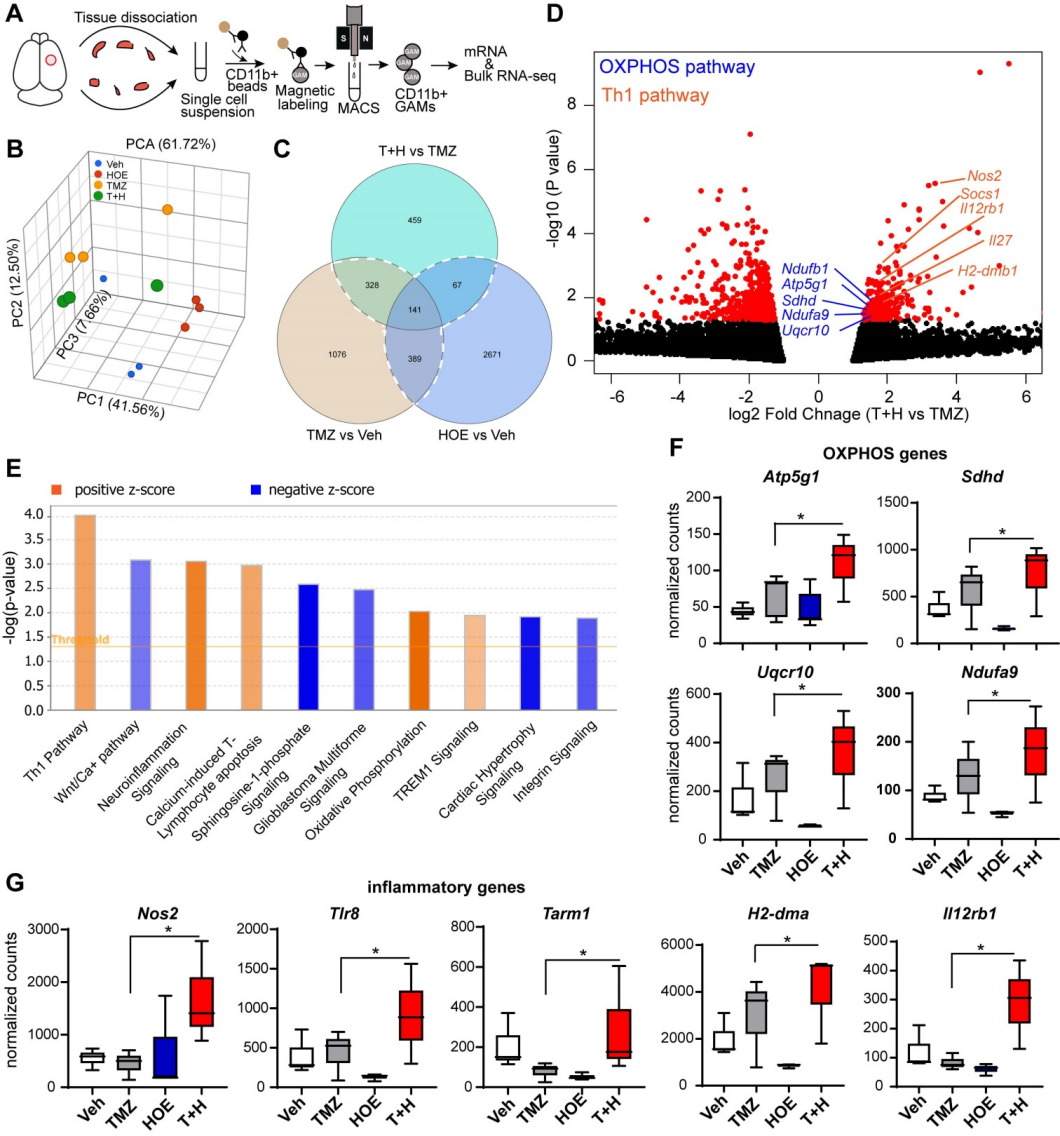
** Bulk RNA-Seq transcriptome analysis of tumor-infiltrating CD11b^+^ cells in response to inhibition of NHE1 in combination with TMZ therapies.** (**A**) Experimental protocol showing isolation of CD11b^+^ cells from GL26 tumor-bearing brain. (**B**) PCA plot showed Veh, HOE, TMZ, and T+H treated brains separated by their top three principal components. (**C**) Venn diagram identified genes of interests for downstream pathway analysis. (**D**) Volcano plots illustrate the gene expression pattern detected (log2 fold change 1.5 and *p*-value < 0.05). (**E**) Enrichment analysis showing significantly altered top canonical pathways by using Ingenuity pathway analysis software. (**F-G**) Box plot showing expression of OXPHOS and proinflammatory genes presented as normalized counts. Data are mean ± SEM, n = 3 mice/group. *p < 0.05 vs indicated.

To better understand how blocking NHE1 protein activity modulates metabolic dysregulation of GAMs, we performed bulk RNA-seq and profiled the transcriptome of isolated tumor-associated CD11b^+^ cells from GL26 glioma tumors that had undergone the four treatment regimens (Veh, HOE642, TMZ or T+H combination therapy, **Fig. 4A**). Principle component analysis (PCA) showed that GAMs transcriptome profiles of the different treatment groups were separated by ~61.7% of their top three principal components (**Fig. 4B**). Since the T+H combination therapy showed increased OXPHOS compared to TMZ monotherapy, here we compared the T+H therapy with the TMZ monotherapy group, and identified a total of 459 genes that were differentially expressed (DEGs) in the T+H treated CD11b^+^ GAMs with a fold change of ≥ 1.5 or ≤ - 1.5 and a *p*-value of ≤ 0.05. Among these genes, 165 were upregulated, and 294 were downregulated (**Fig. 4C-D**). Enrichment analysis using Ingenuity Pathway Analysis (IPA) software shows that Th1 and OXPHOS pathways were among the top 10 pathways altered specifically in the T+H combination therapy-treated GAMs (**Fig. 4E**). In particular, the expression of several genes of the electron transport chain (ETC) was increased by ~1.6-fold in the T+H combination-treated GAMs, such as *Ndufa9, Sdhd, Uqcr10, and Atp5g1* (**Fig. 4D, F**). These genes are involved in the formation of ETC complex I, II, III, and V 24. Expression of several key genes of the Th1 inflammatory pathway (*Nos2, Il12rb1, H2-dma*) was significantly increased in the T+H treated GAMs (by ~2-fold**, Fig. 4D, G**). In addition, *Tlr8* and *Tarm1*, involved in macrophage inflammation 25, 26, were also upregulated in the T+H treated GAMs (~2-fold, **Fig. 4G**). Unsupervised hierarchical clustering confirms these findings where it shows a number of genes displayed altered expression between different treatment groups. *Atp5g1*, *Uqcr10*, *Ndufa9*, *Nos2*, *TLR8*, *H2-Dma* are among the upregulated genes (**Fig. S4**). These genes were significantly upregulated in the T+H treated CD11b^+^ GAMs compared to TMZ treatment. They are involved in OXPHOS and inflammation as shown in **Fig. 4D**. The expression of these key OXPHOS or inflammatory genes in GAMs was further validated by qRT-PCR. We detected a 3-12-fold increase of *Nos2, Tlr8, Tarm1, H2-dma,* and* Il12rb1* gens (*p* < 0.05, **Fig. S5A**) and a 4-15-fold increase of *Atp5g1, Sdhd and Ndufa9* genes in the T+H treated group (*p* < 0.05, **Fig. S5B**). These changes have been reported previously to promote M1 polarization of macrophages 27. Our study provides the first line of evidence that the T+H combination therapy rewired the glucose metabolism of GAMs and polarized to proinflammatory phenotypes.

### Selective deletion of* Nhe1* in Cx3cr1^+^ *Nhe1 KO* mice stimulates glioma tumor immunity in response to the TMZ plus anti-PD-1therapy

Most of the preclinical studies of anti-PD-1 immune checkpoint blockade therapy were done using immunogenic GL-261 glioma with high mutational load 28, 29, resulting in prolonged survival. However, these positive outcomes of immune checkpoint blockade therapy in murine GBM models were not translated into clinical trials; no superior progression-free survival was observed in glioma patients treated with anti-PD-1 monoclonal antibody 2. Therefore, we used the non-immunogenic SB28-GFP gliomas which recapitulate certain clinical features of human GBM in testing immune checkpoint blockade therapy and immune cell profiling.

**Figure 5:**
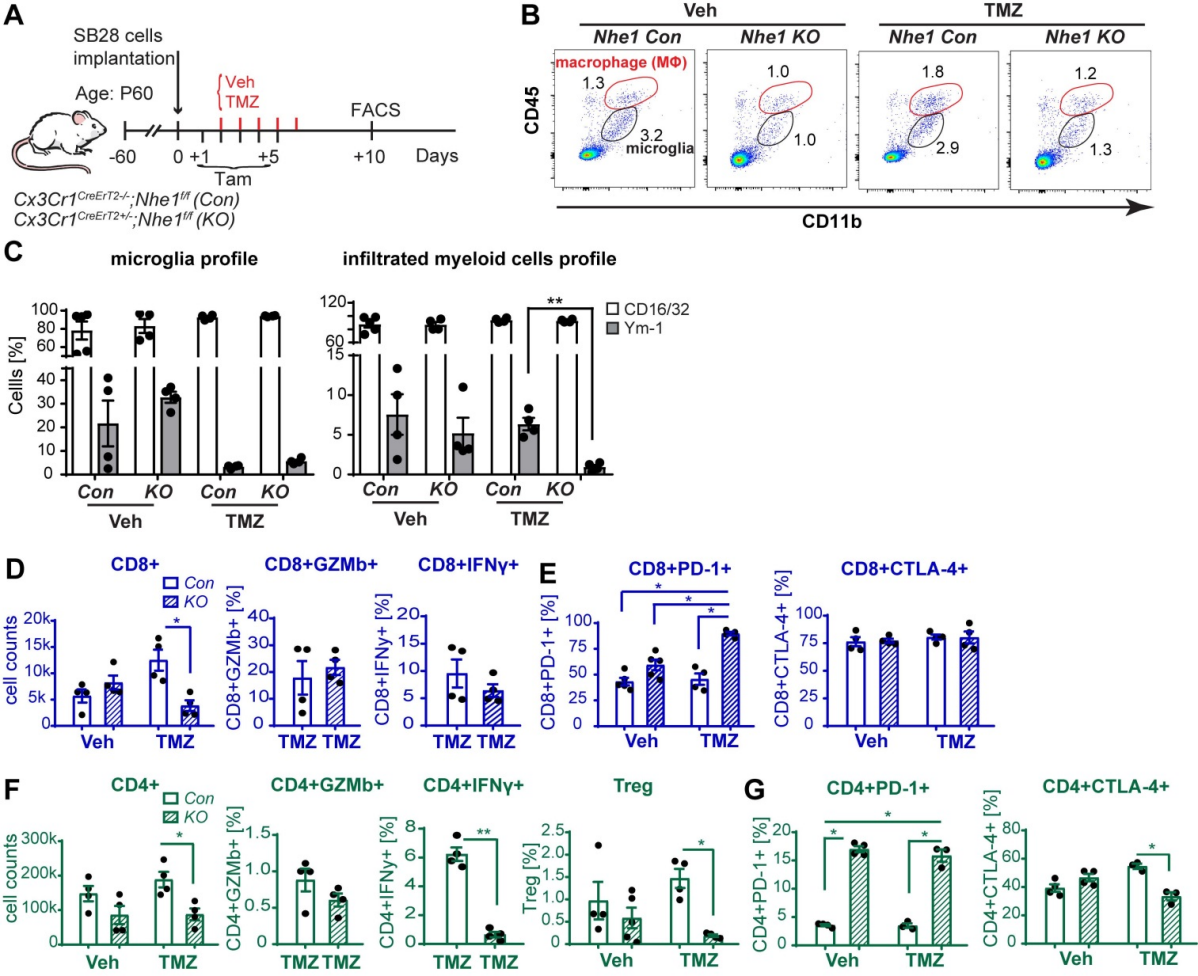
** Selective deletion of* Nhe1* in CX3CR1^+^*Nhe1 KO* mice altered glioma tumor immunity.** (**A**) Tamoxifen-induced selective knockout of *Nhe1* in *Cx3Cr1-Cre^ERT2+/-^*; *Nhe1^f/f^* mice (*Nhe1 KO*). Tam*-treated Cx3Cr1-Cre^ERT2-/-^;Nhe1^f/f^* were used as control group (*Con*). SB28-GFP cells were intracranially transplanted in *Con* and *Nhe1 KO* mice at postnatal 60 days (P60). Mice were treated with either Veh or TMZ. (**B**) Representative FACS gating strategy of microglia (CD11b^+^/CD45^low-medium^) or infiltrated myeloid cells (CD11b^+^/CD45^hi^). (**C**) Inflammatory profile of CD11b^+^/CD45^low-medium^ and CD11b^+^/CD45^hi^ population. (**D**) Changes of infiltrated CD8^+^, CD8^+^GZMb^+^ and CD8^+^IFNγ^+^ profiles. (**E**) Percentages of PD-1^+^ and CTLA-4^+^ cells in the CD8^+^ T-cell populations. (**F**) Changes of CD4^+^, CD4^+^GZMb^+^, CD4^+^IFNγ^+^ and CD4^+^CD25^+^FoxP3^+^ (Treg) profiles and (**G**) Percentages of PD-1^+^ and CTLA-4^+^ cells in the CD4^+^ T-cell populations. Data are mean ± SEM, n = 4-5 mice/group. *p < 0.05 vs indicated.

We further tested our hypothesis by assessing the impact of selective deletion of *Nhe1* in Cx3cr1^+^ cells on changes of glioma immunity in a nonimmunogenic SB28 GBM model which recapitulates key characteristics of human GBM including low mutational load, a factor believed to be responsible for immune checkpoint therapy resistance 30. **Fig. 5A-B** illustrates experimental protocol and analysis of GAMs profiles from *Nhe1 Con* or *Nhe1 KO* mice. The specificity of *Nhe1* deletion was confirmed previously staining for NHE1 protein in IBA^+^ cells 22. A detailed flow cytometry gating strategy is shown in **Fig. S8**. TMZ monotherapy did not change the percentage of proinflammatory CD16/32^+^ and tolerogenic Ym-1^+^ GAMs in* Nhe1 Con* mice **(Fig. 5C)**. In contrast, TMZ treated *Nhe1 KO* mice displayed nearly abolishing Ym-1^+^ myeloid cell infiltration (~150-fold reduction, *p* < 0.05, **Fig. 5C**). These findings suggest that when mice are treated with TMZ, NHE1 protein in GAMs is involved in their infiltration and protumoral, anti-inflammatory polarization.

**Figure 6:**
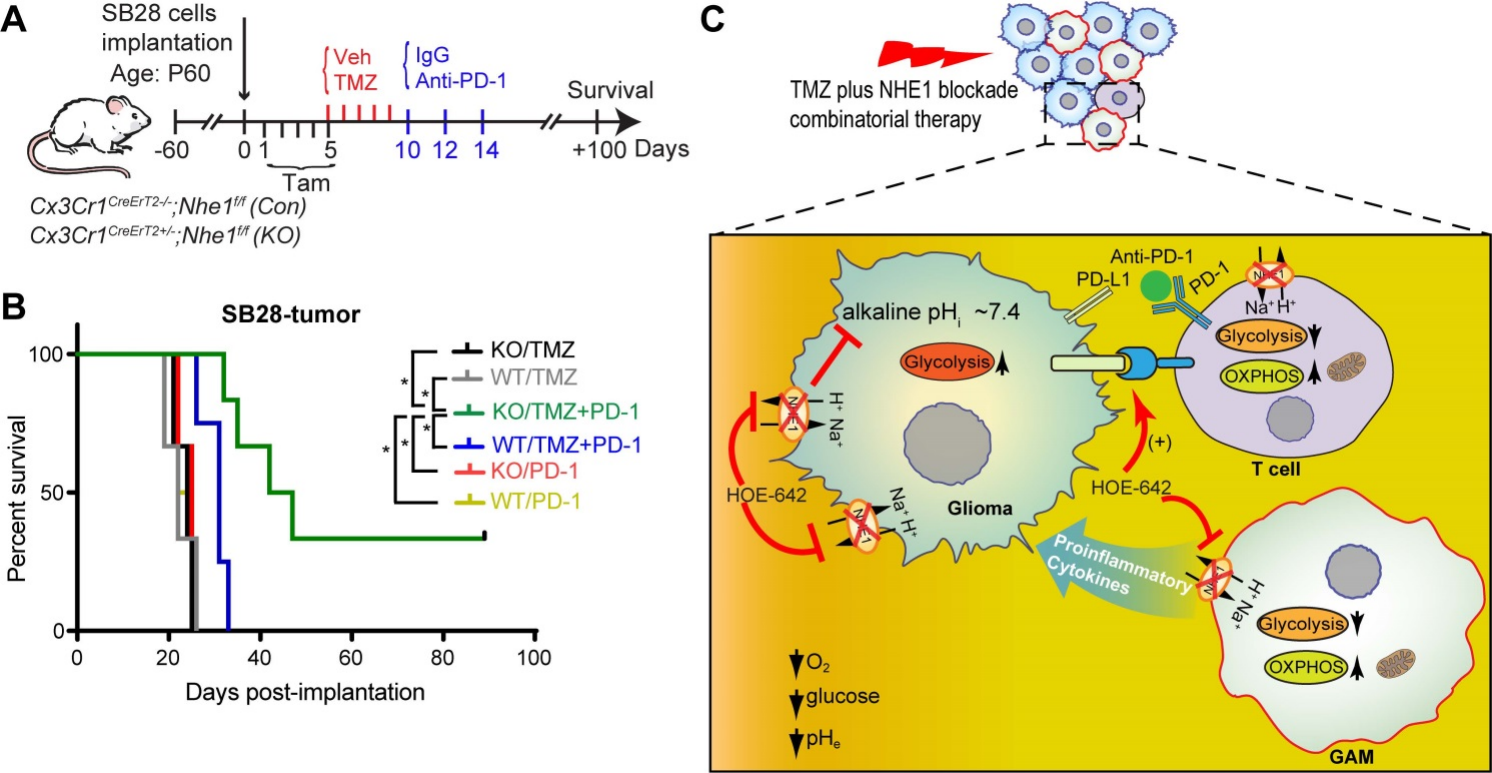
** Selective deletion of* Nhe1* in CX3CR1^+^*Nhe1 KO* mice prolonged survival in response to TMZ and anti-PD-1 combinatorial regimen.** (**A**) Experimental protocol. *Nhe1 Con* or *KO* mice implanted with SB28 tumors were treated with TMZ at 5-9 d.p.i or TMZ followed by anti-PD-1 at 10, 12, and 14 d.p.i. (**B**) Kaplan-Meier survival curve of SB28 tumor implanted mice. n = 4-6 mice/group, *p < 0.05 vs. indicated. (**C**) Summary cartoon showing the role of NHE1 in developing immunosuppressive GBM TME.

We then assessed functional profiles of tumor-infiltrated CD4^+^ and CD8^+^ T-cells and their expression of immune checkpoint molecules (PD-1 and CTLA-4). **Fig. 5D, F** show that similar tumor infiltrated CD4^+^ and CD8^+^ counts were detected in tumor-bearing *Nhe1 Con* and *Nhe1 KO* mice from the Veh-control groups. TMZ monotherapy increased tumor infiltration of CD4^+^ and CD8^+^ T-cells in the *Nhe1 Co*n mice by ~ 2-fold (**Fig. 5D, F**), but, not in the *Nhe1 KO* mice. The *Nhe1 KO* mice showed both reduced CD4^+^IFNγ^+^ as well as reduced CD4^+^CD25^+^FoxP3^+^ Tregs infiltration in response to TMZ monotherapy (*p* < 0.05, **Fig. 5F**). However, in response to TMZ treatment, the T-cells in *Nhe1 KO* mice were accompanied with a simultaneous increase of CD4^+^PD-1^+^ and CD8^+^PD-1^+^ cell populations (*p* < 0.05) and a decrease of CD4^+^CTLA-4^+^ cell counts (~20%, *p* < 0.05, **Fig. 5E, G**). Additionally, TMZ treatment showed a ~3-fold increase of PD-L1 expression in *Nhe1 KO* mice (**Fig. S6**). These data imply that NHE1 protein in GAMs plays a role in modulating the immunosuppressive TME, especially in response to TMZ therapy. Considering the upregulated PD-1 expression in tumor-infiltrating CD4^+^ and CD8^+^ T-cells of *Nhe1 KO* mice, we tested whether these mice were more sensitive to TMZ plus anti-PD-1 combinatorial therapy. Age-matched male and female *Nhe1 Con* and *Nhe1 KO* mice implanted with immunosuppressive SB28 cells were treated with TMZ or TMZ plus anti-PD-1 antibodies (**Fig. 6A**). To maximize research efficiency using transgenic mice, we used both male and female mice in our study in **Fig. 6B**. We did not detect any differences in the survival of male and female mice (**Fig. 6B**). The median survival of male (n = 12) mice was 26 days and female (n = 11) mice was 28 days (*p* = 0.4, log-rank test, data not shown). TMZ or anti-PD-1 monotherapy led to similar median survival in the *Nhe1 Con* mice and *Nhe1 KO* mice (**Fig. 6B**). However, TMZ plus anti-PD-1 therapy significantly prolonged the median survival of *Nhe1 KO* mice but not *Nhe1 Con* mice (**Fig. 6B**, *p* < 0.05). These findings strongly suggest that NHE1 protein in Cx3cr1^+^ GAMs plays a role in regulating glioma immunity and its deletion sensitizes GBM to anti-PD-1 plus TMZ combinatorial therapy.

## Discussion

In this study, by using the ^1^H/^19^F MRI with Vsense (VS1000H DM-red), among the four treatment regimens, we detected the highest IBA-1^+^ myeloid cell accumulation in GBM tumors in response to the TMZ plus NHE1 inhibitor HOE642 (T+H) combination therapy. Immunofluorescence staining illustrated localization of the VS1000H (DM-red) particles in tumor-infiltrating IBA-1^+^ cells and a small fraction of CD8^+^ T-cells. The perfluorocarbon nanoemulsion is capable of labeling both the myeloid and lymphoid cells *in vitro* and taken up by immune cells via endocytosis/phagocytosis 31. However, the major share of PFC is taken up by monocytes due to their larger cytoplasm, compared to T- and B-lymphocytes 32. We speculate that the increased Vsense signals in the tumor-infiltrated IBA-1^+^ cells largely reflect the infiltration of myeloid cells which took up VS1000H. Therefore, these data indicate that the T+H combination therapy enhances anti-tumor immunity by stimulating the infiltration of inflammatory myeloid cells and CD8^+^ T-cells in GBM tumors. Infiltration of immune cells into the tumor has been used to predict the response of immunotherapy which harnesses patients' adaptive and innate immune systems 33. Flow cytometry and immunohistochemistry are common methods to characterize GAMs. ^19^F MRI has been recently used to detect macrophage infiltration into tumors induced by GLV-1h68 virus vaccine-mediated stimulation of innate immunity in lung and breast cancer 34. In addition to myeloid cells and CD8^+^ T-cells, increased tumor infiltration of dendritic cells in GBM tumors has been detected by ^1^H/^19^F MRI with Vsense in response to dendritic cell vaccination 35. Whether the T+H combination therapy stimulates the tumor immunity in part by enhanced dendritic cell infiltration remains to be investigated. Emerging evidence suggests that GAMs increase Warburg aerobic glycolysis, which enables GAMs to survive in the unfavorable, nutrient- and oxygen-deprived TME 9. Glucose metabolism is necessary for increased cytokine production of GAMs 36. In this study, among four treatment regimens, we observed that aerobic glycolysis of CD11b^+^ GAMs was decreased and mitochondrial OXPHOS was increased with the T+H combinatorial therapy, especially compared with TMZ monotherapy. Concomitantly, using bulk RNA-seq and qRT-PCR of GAMs, we detected increased expression of OXPHOS genes *Ndufa9, Ndufb1, Sdhd, Uqcr10, and Atp5g1* in the T+H combination therapy group, which are involved in the formation of ETC complex 24. Transcriptome analysis of CD11b^+^ GAMs also showed increased expression of proinflammatory genes such as *Nos2, Tlr8, Il12rb1, Tarm1, and H2-dma* genes. Taken together, the T+H combination therapy promotes anti-tumor polarization of the GAMs and reprograms their glucose metabolism via increasing OXPHOS and suppressing glycolysis. Our findings are consistent with others that increased mitochondrial OXPHOS of macrophages by CpG oligonucleotide is essential for their anti-tumor response in a mouse model of pancreatic ductal adenocarcinoma 10. In addition, hypoxia, low glucose, high acidity, and high lactate metabolic microenvironment landscape in GBM tumors inhibit T-cell survival, proliferation, and function 7. New evidence suggests that stimulating mitochondrial activation of T-cells can enhance their antitumor activity with increased memory function 37. We detected significantly stimulated glucose uptake and mitochondrial mass of tumor-infiltrating T-cells in mice treated with the T+H combinatorial therapy. Higher mitochondrial mass of CD8^+^ T-cells is associated with increased IFN-γ production 38. Consistently, we have previously shown that blocking NHE1 activity increased numbers of CD8^+^IFN-γ^+^ cells 15. Therefore, the pharmacological blockade of NHE1 activity in combination with TMZ may stimulate anti-tumor immunity in part by enhancing the OXPHOS metabolism of T-cells. Approaches to enhance the GBM immunity by depletion of immunosuppressive macrophages or with CSF-1R inhibitor PLX3397 showed limited efficacy in a preclinical GBM murine model 39 or a clinical trial of recurrent GBM patients 40. This indicates the necessity of developing alternative approaches to reprogram TME. The findings of this study suggest that blunting NHE1 protein function is a novel approach to increase the OXPHOS metabolism of GAMs and T-cells and enhance their anti-tumor function.

In this study, we used the immunogenic GL26 glioma model 41 and the poorly immunogenic SB28 glioma model featured with low MHC-I expression and CD8^+^ T-cell infiltration 30. Previously we have shown that TMZ treatment increased NHE1 protein expression in GL26 and SB28 glioma cells *in vivo* 14. Others have shown that high NHE1 expression leads to the development of acidic TME in breast cancers 42, 43. In this study, we speculate that T+H combination treatment dampened the increased NHE1 activity resulting from TMZ treatment and reversed the acidic TME. Interestingly, in *Nhe1 KO* mice, TMZ monotherapy decreased the immunosuppressive Ym-1^+^ GAMs, Tregs, and T-cells but increased PD-1 expression on T-cells. Taking advantage of this altered immunity in* Nhe1 KO* mice, we employed a combinatorial regimen of TMZ (50 mg/kg/day) and anti-PD-1 therapy for better outcomes. Indeed, the combinatorial therapy significantly prolonged the survival of* Nhe1 KO* mice but not of *Con* mice. Consistent with our findings, it has been reported in the GL26 GBM mouse model that TMZ (100 mg/kg) plus Ad-TK+Ad-Flt3L gene therapy prolonged the animal survival despite the reduced T-cell infiltration 44. In contrast, no superior survival of GL261 tumor-bearing animals was shown with a 50 mg/kg/day dose of TMZ+anti-PD-1 therapy, which was attributed to the overexpression of the immune checkpoint pathway genes 45. MGMT methylation is known to be associated with TMZ response with a favorable prognosis 46. Additionally, a recent report shows that TMZ-induced ER stress is dependent on MGMT deficiency which potentiates CD47 blockade and increases glioma cell phagocytosis 47. GL26 cells had no MGMT expression 48, and MGMT expression of SB28 is unknown 49, therefore, whether the anti-tumor effect of TMZ in *Nhe1 KO* mice is dependent on MGMT methylation status or TMZ-induced ER stress remains to be investigated. The immune checkpoint blockade therapy with nivolumab failed to prolong the overall survival of recurrent GBM patients due to the non-immunogenic nature of GBM with limited effector immune cell infiltration 3. In this context, our new findings indicate that TMZ combined with selective *Nhe1* deletion in GAMs effectively stimulates tumor immunogenicity by increasing tumor infiltration of immune cells and elevated PD-1 expression. Therefore, NHE1 protein in GAMs plays a critical role in developing immunosuppressive GBM TME and its blockade emerges as a strategy for reinvigorating T-cells anti-tumor function in combination with TMZ and anti-PD-1 therapy.

The function of TMZ as an immune modulator combined with immunotherapy has been investigated. TMZ monotherapy has been reported to cause lymphodepletion 50 and increase immunosuppressive Tregs 51 in GBM patients. In our study, we detected that TMZ monotherapy stimulates Th2 polarization of tumor-infiltrating CD11b^+^ GAMs (**Fig. S7**) in contrast to the anti-tumor activation profile of GAMs in the T+H combinatory therapy group. On the other hand, TMZ plus dendritic cell vaccine prolonged the median survival of GBM patients with increased CD4^+^ T-cell activity 52. TMZ plus IL-2Rα antibody reduced tumor volume in the mouse OT-1 GBM model 51. In our study, TMZ treatment in *Nhe1 KO* mice nearly abolished the Ym-1^+^ tolerogenic GAMs. Taken together, our study with *Nhe1* deletion in GAMs or pharmacological NHE1 blockade in conjunction with TMZ therapy modulates TME by reducing tumor-promoting tolerogenic GAMs.

NHE1 inhibitor cariporide (HOE642) has been clinically tested for the treatment of cardiac disease and ischemia reperfusion injury 53. In a Phase III clinical trial, cariporide significantly reduced the incidence of death or myocardial infarction in post-operative patients 54. However, patients received cariporide developed ischemic embolic stroke, a condition that may arise from the prolonged infusion of cariporide, reduced procoagulant response, and the stimulation of platelet function 55. A new selective NHE1 inhibitor, N-[4-(1-acetyl-piperidin-4-yl)-3-trifluoromethyl-benzoyl]-guanidine (BIX) has been identified with high selectivity against NHE1 (IC50, 6 nM) in comparison with cariporide (IC50, ~ 0.03-3.4 µM), and potentially lower effects on platelets 56 proved effective in the prevention of ischemic myocardium damages in a rat myocardial infarction model 57. Therefore, pharmacological NHE1 inhibition represents a well-tolerated potent anti-cancer drug with high specificity and low toxicity 58. However, more studies are needed to validate existing inhibitors or to develop novel NHE1 inhibitors for anti-cancer therapy.

In summary, we report that pharmacological blockade of NHE1 protein in combination with TMZ increases myeloid cell tumor infiltration, stimulates glucose uptake and mitochondrial glucose metabolism in GAMs and tumor-infiltrating T-cells in a mouse GBM model (**Fig. 6C**). Bulk RNA-seq transcriptome profiling revealed the upregulation of OXPHOS genes and proinflammatory genes in these GAMs. Selective deletion of *Nhe1* in microglia/myeloid cells revealed their reduced tolerogenic activation and reduced the number of immunosuppressive Tregs but increased the PD-1 expression on CD4^+^ and CD8^+^ T-cells. Importantly, TMZ combined with anti-PD-1 therapy prolonged median survival of *Nhe1 KO* mice but not *Nhe1 Con* mice. Taken together, these findings suggest that NHE1 protein plays an important role in promoting immunosuppressive TME by dysregulating glucose metabolism of GAMs and tumor-infiltrating T-cells.

## Supplementary Material

Supplementary figures and tables.

## Abbreviations

CGGA: Chinese genome glioma atlas
ECAR: extracellular acidification rate
ETC: electron transport chain
GAM: glioma-associated microglia/myeloid cells
GBM: glioblastoma
IBA-1: ionized calcium-binding adapter molecule 1
*KO*: knockout
MDSC: myeloid derived suppressor cell
MRI: magnetic resonance imaging
NHE1: sodium/hydrogen exchanger 1
OCR: oxygen consumption rate
OXPHOS: oxidative phosphorylation
PD-1: programmed cell death protein 1
PD-L1: programmed death ligand
PFC: perfluorocarbon
TMZ: temozolomide
TME: tumor microenvironment
