# Supplementary material for: Blocking NHE1 stimulates glioma tumor immunity by restoring OXPHOS function of myeloid cells
Source: Theranostics. 2021 Jan 1;11(3):1295–309. doi: 10.7150/thno.50150 (PMC7738877; doi:10.7150/thno.50150)
Supplement: Supplementary file 1 — Supplementary figures and tables. [file thnov11p1295s1.pdf]

Blocking NHE1 stimulates glioma tumor immunity by restoring OXPHOS function of myeloid cells

Md Nabiul Hasan[1], Lanxin Luo[1], Dawei Ding[1], Shanshan Song[1], Mohammad Iqbal H. Bhuiyan[1], Ruijia Liu[1,6], Lesley M. Foley[2], Xiudong Guan[1], Gary Kohanbash[3], T. Kevin Hitchens[2, 4], Maria G. Castro[5], Zhongling Zhang[6*], Dandan Sun[1,7*]

[1]Department of Neurology and Pittsburgh Institute for Neurodegenerative Disease, University of Pittsburgh, Pittsburgh, PA 15213; [2]Animal Imaging Center, University of Pittsburgh, Pittsburgh, Pennsylvania, 15213; [3]Department of Neurological Surgery, University of Pittsburgh, Pittsburgh, PA 15213; [4]Department of Neurobiology, University of Pittsburgh, Pittsburgh, Pennsylvania, 15213; [5]Department of Neurosurgery, University of Michigan Medical School, Ann Arbor, MI, USA; [6]Department of Neurology, First Affiliated Hospital, Harbin Medical University, Harbin, Heilongjiang, China; [7]Veterans Affairs Pittsburgh Health Care System, Geriatric Research, Educational and Clinical Center, Pittsburgh, Pennsylvania

Corresponding authors:
Dandan Sun, M.D., Ph.D. Department of Neurology, University of Pittsburgh Medical Center, USA. E-mail address: sund@upmc.edu

Zhongling Zhang, M.D, Ph.D, The First Affiliated Hospital, Harbin Medical University, Department of Neurology, China. Email address: zhangzhongling@outlook.com

Materials and methods

Temozolomide (TMZ) (#T2577), tamoxifen (Tam) (#T5648) and dimethyl sulfoxide (DMSO) (#D2650) were purchased from Sigma-Aldrich (St. Louis, MO). Dulbecco's modified Eagle medium (DMEM/HEPES, #12430-054), Penicillin/streptavidin (#15240062) were from Gibco (Carlsbad, CA). Fetal bovine serum (FBS) (#S11150H) was purchased from Atlanta Biologics (Flowery Branch, GA). Fixation/permeabilization kit (#00-5523-00), and MitoTracker (#M22426), PE-GZMb (#12-8898-80) were obtained from Invitrogen (Carlsbad, CA). Neural tissue dissociation kit (#130-092-628) and CD11b microbead (#130-093-634) were obtained from Miltenyi Biotech (Gladbach, Germany). 2-NBDG (2-(*N*-(7-Nitrobenz-2-oxa-1,3-diazol-4-yl)Amino)-2-Deoxyglucose) (#11046) was purchased from Cayman Chemicals (Ann Arbor, Michigan). Percoll (#17-0891-01) was purchased from GE Healthcare (Upsala, Sweden). Anti-mouse PD-1 (#BE0146) and isotype IgG2a (#BE0089) were obtained from BioXCell (West Lebanon, NH). The mouse antibody against NHE1 (#sc-136239) was from Santa Cruz Biotechnology (Dallas, TX). BUV395-CD11b (#563553), BV510-CD45(#103138), PE-Cy5-CD8a(#100710), Apc/Cy7-CD4(#100414), PE-FoxP3(#12-5773-82), PE-PD-1(#114118) and PE-Cy7-CTLA-4 (#106314) were obtained from Biolegend (San Diego, CA). APC-IFNγ (#1773181) and eFluor 450-CD16/32 (#48-0161-82) were purchased from eBioscience (San Diego, CA); PE-Ym1 (#ab211621) was from Abcam (Cambridge, MA). Rabbit anti-ionized calcium-binding adapter molecule 1 (IBA-1) (#CTF4377) was from Wako (Richmond, VA), rat anti-CD8 (#ab22378) was from Abcam Ltd (Cambridge, MA) and Goat anti-rabbit Alexa Fluor 633 secondary antibody was from Thermo Scientific. VS1000H DM (Texas Red) was purchased from Celsense Pittsburgh. Vectashield mounting medium for fluorescence was from Vector Laboratories (Burlingame, CA). RNeasy Plus Mini Kit (#74234) was purchased from Qiagen

(Hilden, Germany). iScript cDNA kit (#1708891) and iTaq Universal SYBR Green (#1725121) were purchased from BioRad (USA). V-Sense was obtained from Celsense, Pittsburgh, PA.

Immunofluorescence staining of post-MRI *ex-vivo* brains

Post $^{1}H/^{19}F$ MRI, fixed brains were equilibrated in 30% sucrose at 4ºC and sectioned as described before [1]. Coronal brain sections (25 µm) were washed with PBS and incubated with blocking solution (10% normal goat serum, 0.5% Triton X-100 in 0.1 M PBS) for 1 h at room temperature and then incubating with rabbit monoclonal anti-IBA-1 (1:200) and rat monoclonal anti-CD8 (1:200) in blocking solution overnight at 4^{0}C. Sections were washed with PBS and incubated with goat anti-rabbit or goat ant-rat Alexa 633 conjugated secondary antibodies. Fluorescence images were captured with an Olympus IX81 confocal microscope under a 40x oil immersion lens. Images were analyzed using Image J (National Institute of Health) software.

Glucose uptake and mitochondrial mass of GAMs and T-cells

Single GAM suspension obtained from MACS was incubated with 30 µM 2-NBDG in RPMI-1640 media supplemented with 2% FBS and 2 mM glutamine for 30 min at 37 ºC. Cells were then simultaneously surface stained with APC-Cy7 CD4, PE-Cy5 CD8, anti-mouse PE-cy7-CD11b, BV510-CD45 antibody and loaded with 30 nM of MitoTracker FM for 30 min at 4 ºC. Data were acquired with a BD LSRII instrument and fluorescent intensity of 2-NBDG and MitoTracker FM were analyzed with Flow Jo (Tree Star) software.

Flow cytometry analysis (FACS) of tumor infiltrated immune cells

Brain tumor tissues were dissociated, and myelin was removed by centrifugation in a gradient of 22% percoll, as described previously [2]. For profiling of GAMs, dissociated cells were stained with anti-mouse APC-CD11b, BV510-CD45, PE-Ym1, eFluor 450-CD16/32. Microglia population ($CD11b^{+}CD45^{low\text{-}medium}$) was separated from the myeloid population ($CD11b^{+}CD45^{hi}$) by flow cytometry with a BD LSRII instrument and analyzed with Flow Jo (Tree Star) software, which has been validated with ~ 90% microglial cells expressing P2RY12 specific marker [2]. For T-Cell profiling, cells were stained with Percp/Cy5.5-CD8a, Apc/Cy7-CD4, APC-CD25, PE-FoxP3, APC-IFNγ, and PE-GZMb. Intracellular staining of FoxP3 was done by permeabilization of PFA fixed cells using fixation/permeabilization kit according to the manufacturer's instruction. For detecting immune checkpoint blocker expression, cells were stained with PE-Cy5 CD8a, Apc/Cy7-CD4, PE-PD-1, and BV421 CTLA-4.

qRT-PCR of tumor isolated $CD11b^{+}$ GAMs

Isolated $CD11b^{+}$ cells (1×10^{6} Cells/sample) were lysed in Qiagen RLT buffer immediately following MACS sorting, and RNA was extracted using the RNeasy Mini kit (Qiagen) according to the manufacturer's instructions. RNA was quantified by measuring absorbance with spectrophotometer ND-1000 (NanoDrop). Reverse transcription was performed using the iScript Reverse Transcription Supermix (Bio-Rad) according to the manufacturer's protocol. All RNA isolated from cell pellets was converted into cDNA. Quantitative RT-PCR was performed using iTaq Universal SYBR Green Supermix (Bio-Rad) on a CFX 96 Touch Real-Time PCR Detection System. Relative gene expression analyses were performed using the $2^{-\Delta\Delta Ct}$ method with

triplicate reactions for each gene evaluated. Primer sequences used are listed in **Supplementary Table S1**.

Figure S1

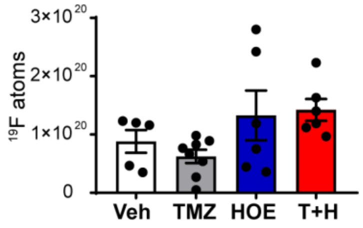

Figure S1: Pharmacological inhibition of NHE1 alone or in combination with TMZ increase ^{19}F signal in the tumor

^{19}F signal intensity as detected by voxel tracking software after VS1000H Texas Red dye was injected into mouse tail vein and ^{19}F MRI was performed. Data are mean ± SEM, n = 5-7 mice/group.

Figure S2

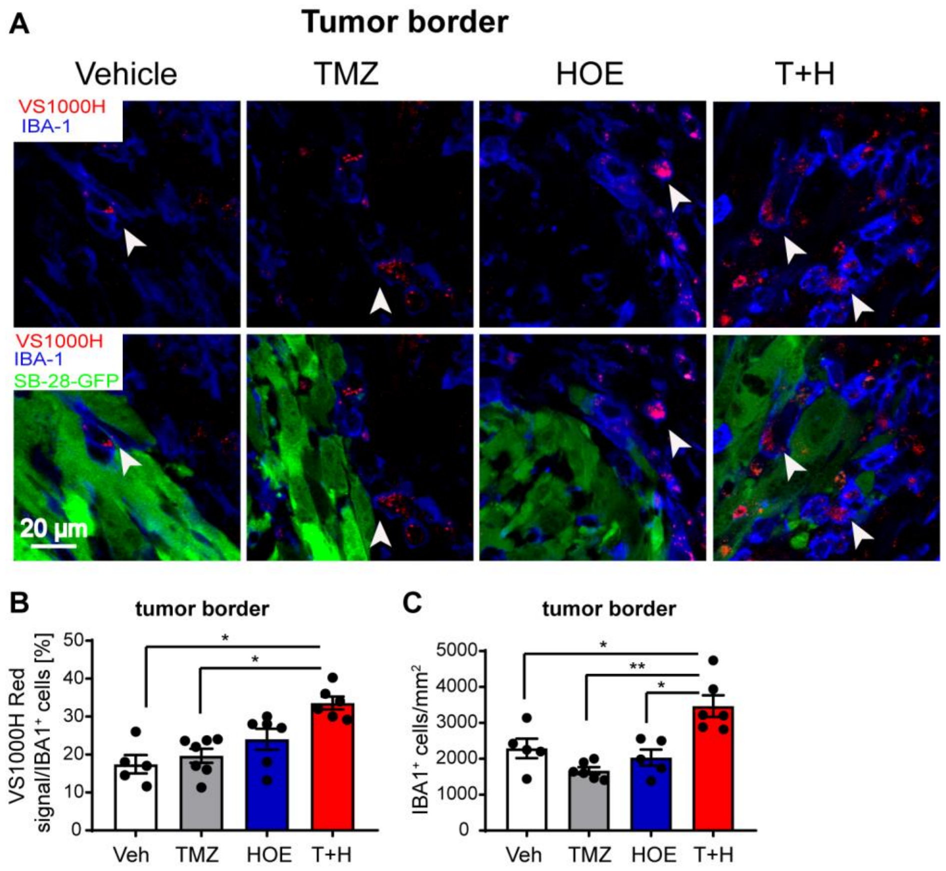

Figure S2. Pharmacological inhibition of NHE1 in combination with TMZ increase IBA-1$^+$ cells in tumor border

(**A**) Representative confocal images of perfluorocarbon (VS1000H, Texas Red, arrowheads) loaded GAMs and IBA1 staining of brain sections collected post-MRI from the same cohort of SB28 glioma bearing mice in **Fig. 1**: Veh-, TMZ-, HOE642-, and T+H-treated mice. (**B**) Summary data of VS1000H dye$^+$/IBA-1$^+$ cells and (**C**) IBA-1$^+$ cells infiltrated into tumor border. Data are mean ± SEM, n = 5-7 mice/group. *$p < 0.05$ vs indicated.

Figure S3

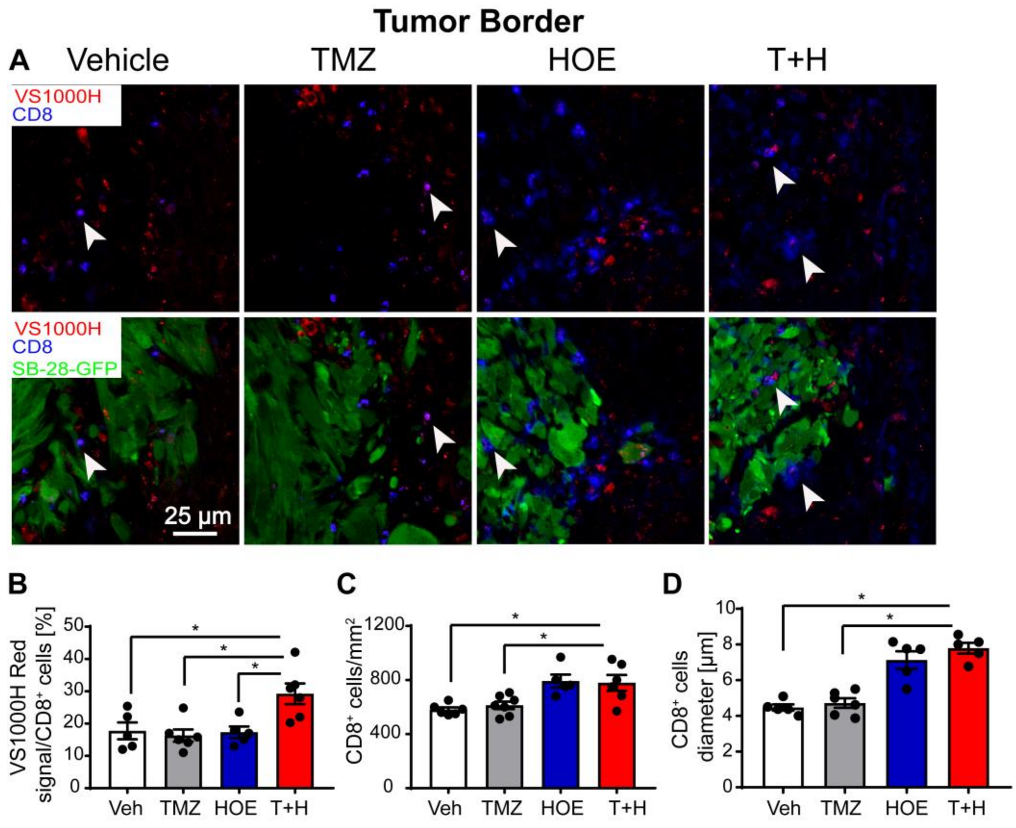

Figure S3. Pharmacological blockade of NHE1 in combination with TMZ therapy increase $CD8^+$ T-cell infiltration

(**A**) Representative confocal images of perfluorocarbon (VS1000H, Texas Red, arrowheads) loaded $CD8^+$ T-cells in the SB28 tumor border collected post-MRI from the same cohort of SB28 glioma bearing mice in **Fig. 1**. (**B**) Summary data of VS1000H $dye^+/CD8^+$ cells and (**C**) $CD8^+$ cells infiltrated in the tumor border. (**D**) The diameter of $CD8^+$ T-cells. Data are mean ± SEM, n = 5-7 mice/group. *$p < 0.05$ vs indicated.

Figure S4

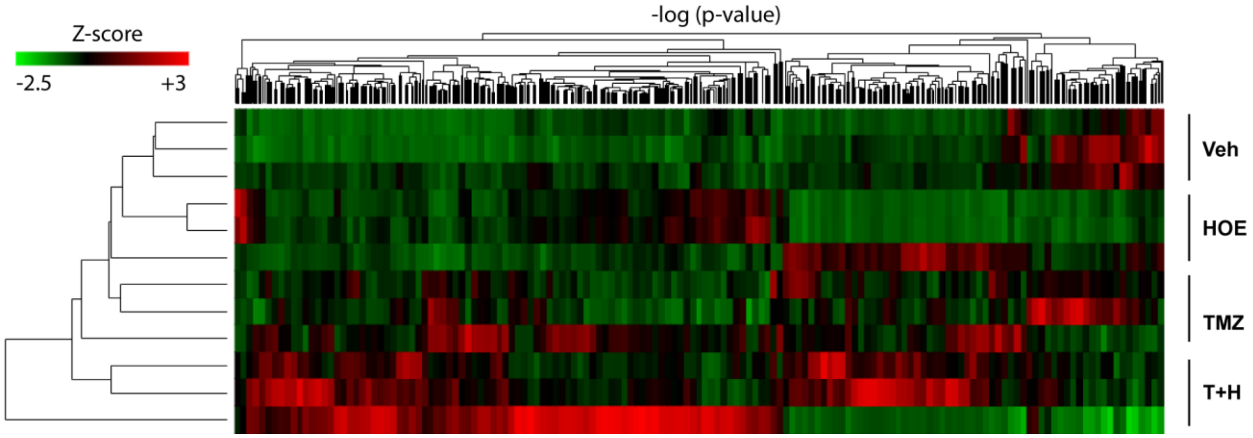

Figure S4. Heat map illustrates up- and down-regulated genes

Unsepervised hierarchical clustering showing DEGs between different treatment groups as displayed in **Fig. 4**. A cutoff fold change of ≤ - 1.5 or ≥ 1.5 and p value < 0.05 were applied.

Figure S5

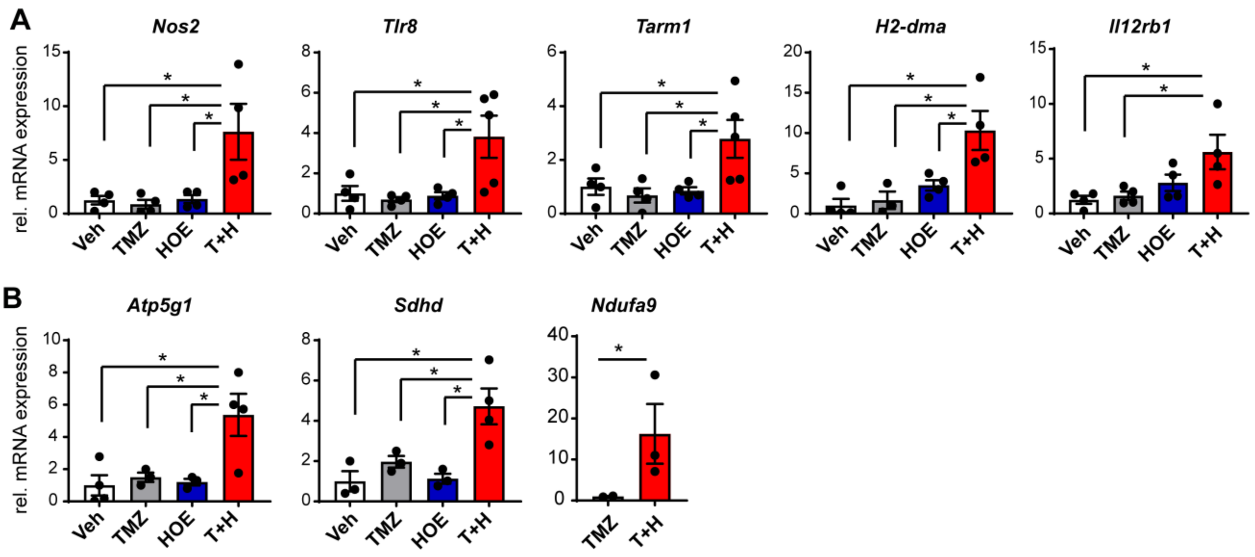

Figure S5. Pharmacological blockade of NHE1 in combination with TMZ therapy increase proinflammatory and electron transport chain genes in CD11b$^+$ cells

(**A**) qRT-PCR quantification of *NOS2, Tlr8, tarm1, H2-dma,* and *Il12rb1* genes and (**B**) *Atp5g1, Sdhd*, and *Ndufa9* genes from SB28 tumor isolated CD11b$^+$ cells. Data are mean ± SEM, n = 3-5 mice/group. *p < 0.05 vs indicated.

Figure S6

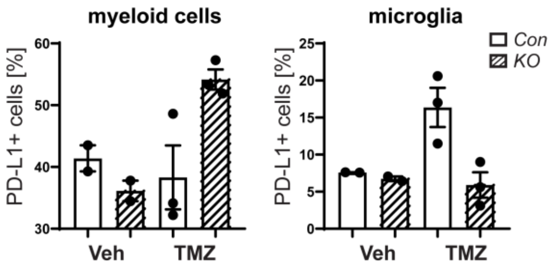

Figure S6. TMZ treatment in CX3CR1^{+} *Nhe1* KO mice increases PD-L1 expression in infiltrated myeloid cells.

Percentage of PD-L1^{+} cells gated within the infiltrated myeloid cells (CD11b^{+}/CD45^{hi}) or microglia (CD11b^{+}/CD45$^{low\text{-}medium}$). Data are mean ± SEM, n = 2-3 mice/group.

Figure S7

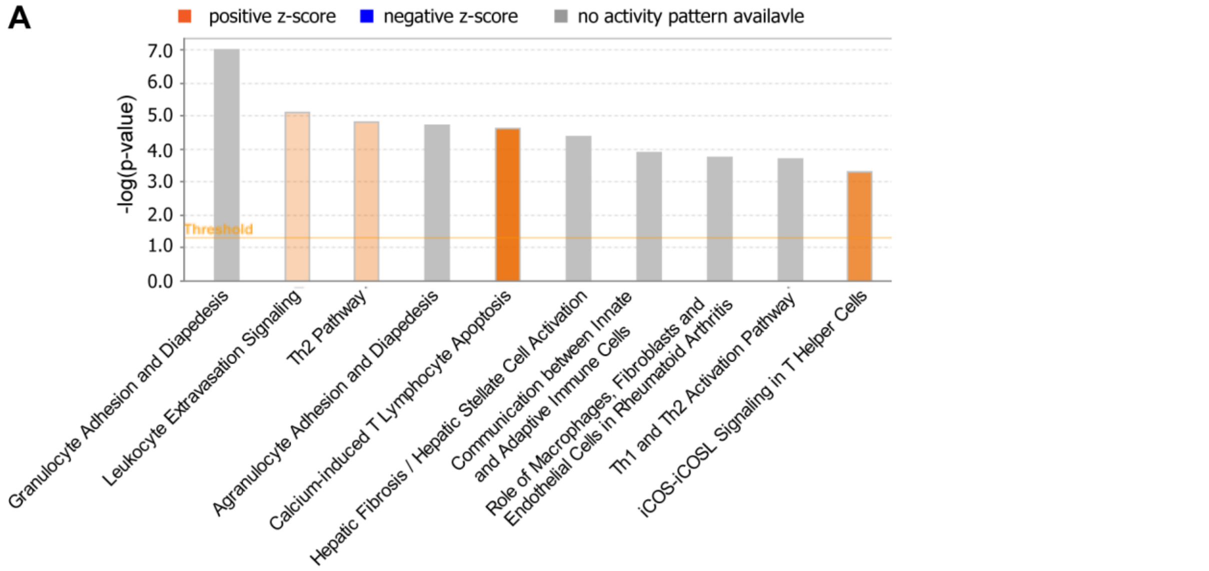

Figure S7. Bulk RNA-Seq transcriptome analysis of tumor-infiltrating CD11b$^+$ cells

(**A**) Enrichment analysis showing significantly altered top 10 canonical pathways by using Ingenuity pathway analysis software using DEGs from TMZ vs Veh treated CD11b$^+$ cells of the same cohort of GL26 tumor bearing mice as shown in **Fig. 4**. No downregulated pathway (negative z-score) was detected in the top 10 altered pathways. (**B**) Box plot displays the expression of Th2 pathway genes in CD11b$^+$ cells. n = 3 mice/group. *$p < 0.05$ vs indicated.

Figure S8

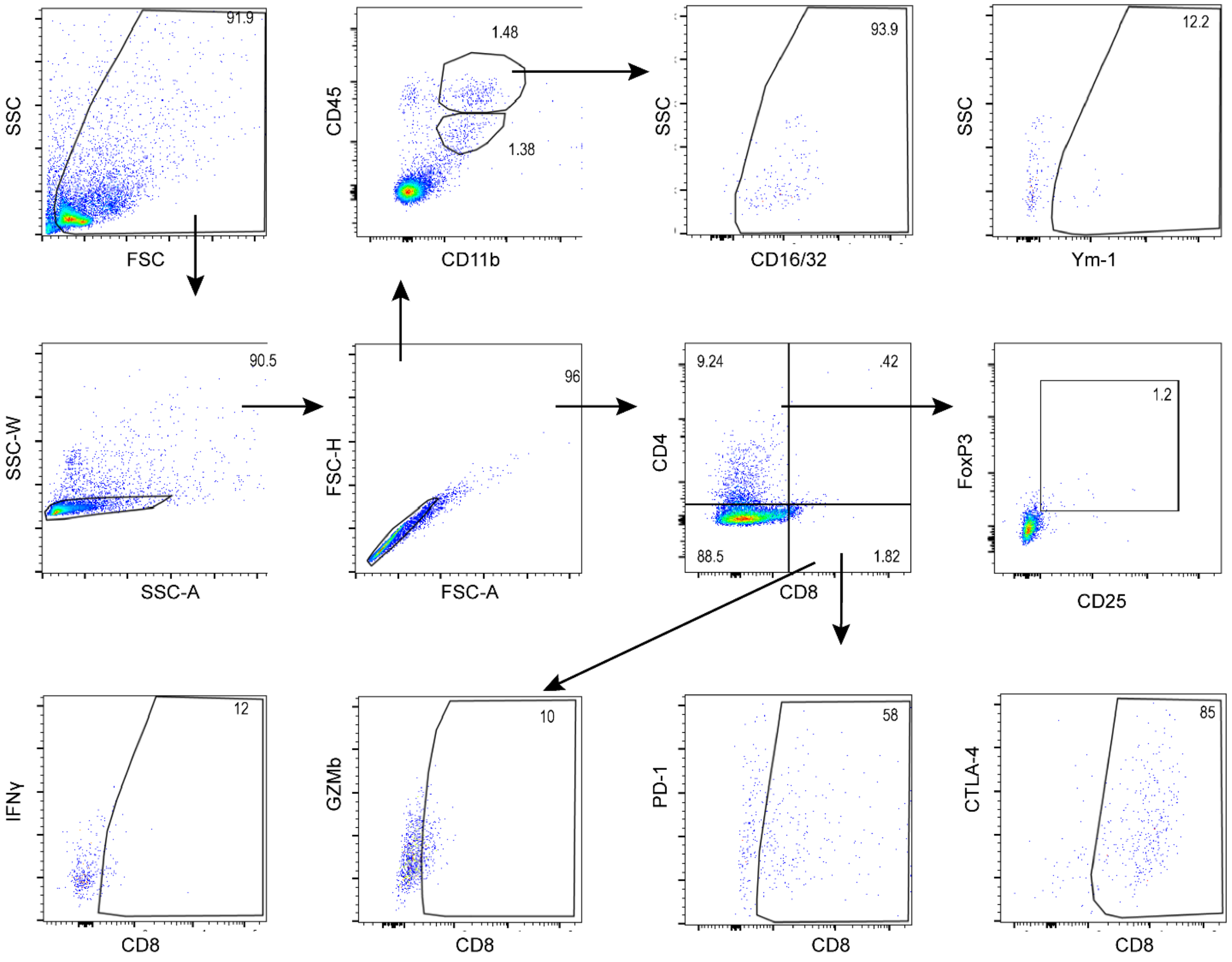

Figure S8. Gating strategy for individual immune cell types of a representative WT (Veh-treated) sample.

Table S1. Primer sequences used in qRT-PCR

Gene	Forward	Reverse
Nos2	GTTCTCAGCCCAACAATACAAGA	GTGGACGGGTCGATGTCAC
Tlr8	CAAACAACAGCACCCAAAT	GGGGGCACATAGAAAAGGTT
Tarm1	ACATTCCTGAAAATGGGTCTCCT	TTCACAGGTGTAGTGTCCGC
H2-dma	TGAGCAGAAGTCAGGAGCTG	GTGGGTCATCCCACAACACT
Il12rb1	GGACCAGCAAACACATCACCTT	CAACGCAGCAGCCATCAC
Sdhd	CCAGCACATTCACCTGTCA	ATCAGCCCCAAGAGCAGAA
Ndufa9	CAT TAC TGC AGA GCC ACT	ATC AGA CGA AGG TGC ATG AT
Atp5g1	AGTTGGTGTGGCTGGATCA	GCTGCTTGAGAGATGGGTTC
Ppia	TGTGCCAGGGTGGTGACTTT	CGTTTGTGTTTGGTCCAGCAT

References

1. Begum G, Song S, Wang S, Zhao H, Bhuiyan MIH, Li E, et al. Selective knockout of astrocytic Na(+) /H(+) exchanger isoform 1 reduces astrogliosis, BBB damage, infarction, and improves neurological function after ischemic stroke. Glia. 2018; 66: 126-44.
2. Guan X, Hasan MN, Begum G, Kohanbash G, Carney KE, Pigott VM, et al. Blockade of Na/H exchanger stimulates glioma tumor immunogenicity and enhances combinatorial TMZ and anti-PD-1 therapy. Cell Death Dis. 2018; 9: 1010.
